# Crowdsourcing Framework for Security Testing and Verification of Industrial Cyber-Physical Systems

**DOI:** 10.3390/s26010079

**Published:** 2025-12-22

**Authors:** Zhenyu Li, Yong Ding, Ruwen Zhao, Shuo Wang, Jun Li

**Affiliations:** 1 School of Computer Science and Information Security, Guilin University of Electronic Technology, Guilin 541004, China; lizhenyu@guet.edu.cn (Z.L.); swang@guet.edu.cn (S.W.); 2 Postdoctoral Research Station in Instrument Science and Technology, Guilin University of Electronic Technology, Guilin 541004, China; 3 Guangxi Academy of Artificial Intelligence, Nanning 530028, China; zhaoruwen@guet.edu.cn; 4 School of Mathematics and Computing Science, Guilin University of Electronic Technology, Guilin 541004, China; 5 China Industrial Control Systems Cyber Emergency Response Team, Beijing 100040, China; ljfigo@126.com

**Keywords:** industrial cyber-physical system, testing, security

## Abstract

With the widespread deployment of Industrial Cyber-Physical Systems (ICPS), their inherent vulnerabilities have increasingly exposed them to sophisticated cybersecurity threats. Although existing protective mechanisms can block attacks at runtime, the risk of defense failure remains. To proactively evaluate and harden ICPS security, we design a distributed crowdsourced testing platform tailored to the four-layer cloud ICPS architecture—spanning the workshop, factory, enterprise, and external network layers. Building on this architecture, we develop a Distributed Input–Output Testing and Verification Framework (DIOTVF) that models ICPS as systems with spatially separated injection and observation points, and supports controllable communication delays and multithreaded parallel execution. The framework incorporates a dynamic test–task management model, an asynchronous concurrent testing mechanism, and an optional LLM-assisted thread controller, enabling efficient scheduling of large testing workloads under asynchronous network conditions. We implement the proposed framework in a prototype platform and deploy it on a virtualized ICPS testbed with configurable delay characteristics. Through a series of experimental validations, we demonstrate that the proposed framework can improve testing and verification speed by approximately 2.6 times compared to Apache JMeter.

## 1. Introduction

With the rise of Industry 4.0, Industrial Cyber-Physical Systems (ICPS) are being rapidly adopted by enterprises to enable intelligent, automated, and data-driven manufacturing. By tightly integrating computing resources, network communication, and physical processes, ICPS can monitor and adjust production activities in real time, reducing manual intervention while improving efficiency, resource utilization, and production flexibility [1]. These systems have become a key enabling technology for smart factories and digital transformation [2]. The global ICPS market is projected to reach 177.5 billion USD by 2030, reflecting its accelerating adoption across sectors such as energy, transportation, logistics, and critical infrastructure [3]. However, as ICPS become increasingly interconnected and shift from isolated internal networks to cloud-connected architectures, they also face increased exposure to cyber threats [4,5]. A notable turning point was the Stuxnet attack, which demonstrated that malware could infiltrate industrial control systems, manipulate cyber-physical operations, and cause real physical damage [6,7]. Since then, securing ICPS has become a priority concern for both industry and governments worldwide.

Despite the adoption of many security mechanisms, existing defenses for ICPS remain largely reactive and insufficient in the face of increasingly sophisticated cyberattacks. As ordinary enterprises connect ICPS to the Internet and cloud platforms, incidents such as the Colonial Pipeline ransomware attack in May 2021 have shown how a single breach can disrupt critical operations and cause significant economic loss [8]. The multilayered architecture of ICPS, spanning hardware, software, edge devices, and networks, introduces numerous potential attack surfaces, especially when combined with cloud services. From a risk perspective, ICPS are exposed to multiple categories of threats, including: network- and communication-layer risks such as eavesdropping, replay, and man-in-the-middle attacks; control-logic and protocol-implementation risks such as malformed command injection and logic bypass; data integrity and availability risks such as false data injection and denial-of-service; and configuration and access-control risks such as weak authentication and privilege abuse. Many ICPS still operate with unauthenticated or unencrypted communication protocols to prioritize performance, making them particularly vulnerable to these threats [9]. Moreover, long-term deployment within internal edge networks often results in overlooked vulnerabilities and insufficient proactive security checks. Attackers can exploit these weaknesses, chaining multiple vulnerabilities together to send incorrect control signals, tamper with physical processes, or bring production to a halt [10]. Therefore, beyond traditional perimeter-based defense, there is an urgent need for proactive, vulnerability-aware security solutions that can detect risks early and prevent attacks before they cause physical or operational damage.

To overcome the lack of proactive security verification mechanisms in industrial cyber-physical systems, this paper develops a distributed crowdsourced testing platform specifically designed for cloud-based ICPS environments. Building upon a four-layer architectural model that spans workshop, factory, corporation, and external network layers, this platform enables security testing across heterogeneous hardware, software, and network environments while simulating realistic production conditions. To address core challenges in distributed ICPS testing, particularly the difficulty of post-test result verification due to asynchronous network delays, we propose a Distributed Input–Output Testing and Verification Framework (DIOTVF) that supports controllable communication delays and multithreaded parallel testing. This framework enables testers to generate test events with precise timing while improving verification accuracy and reproducibility. Based on the framework, we further design and implement a multithreaded testing architecture capable of adjustable delay control, significantly improving test verification speed over existing tools such as Apache JMeter. The proposed platform allows enterprises to proactively detect potential vulnerabilities before system deployment or during continuous operations, making it a practical tool for security assurance in industrial intelligence scenarios.

In addition, this paper uses the term crowdsourced testing in a practical engineering sense. The platform is designed to coordinate a large number of geographically distributed testing agents (e.g., in-house QA teams from different plants, external security testers under contract, and automated test nodes deployed in different networks) that pull test tasks from a shared task pool and report their results back to the platform, instead of relying on a single centralized executor. We do not address incentive schemes or workforce management; rather, we abstract the “crowd” as a set of authenticated but mutually untrusted test nodes that can be dynamically added or removed. Under this abstraction, crowdsourcing is necessary mainly for scaling ICPS security testing across many factories, configurations, and network locations, and for increasing test diversity beyond what a single testbed can provide. Moreover, we use “testing” to refer to the process of injecting stimuli into the system under test, and “verification” in a narrower, runtime sense, namely checking whether the responses observed on distributed outputs conform to the expectations encoded in the test templates. We do not claim formal verification of all system requirements. Within this architectural view, the DIOTVF presented in the remainder of this paper should be understood as the core testing engine of the crowdsourced platform: each registered agent runs instances of the sender and verifier components, while the central service maintains the shared test object pool and orchestrates task assignment and aggregation of verification statistics.

The main contributions of this paper are as follows:

- A distributed ICPS testing and verification architecture tailored for crowdsourced execution. We design a testing and verification architecture for a four-layer cloud-based ICPS that decouples the system under test from the testing crowd via standardized test and verification ports, a shared test object model, and a dynamic test–task management mechanism. This allows heterogeneous testing agents deployed in different networks to exercise the same ICPS deployment without requiring intrusive instrumentation of industrial controllers.
- A delay-aware input–output testing and verification framework for distributed ICPS. We propose a protocol-agnostic, template-driven testing and verification framework that explicitly models spatially distributed inputs and outputs, maintains a hash-based pool of in-flight test objects, and tolerates random communication delays and out-of-order responses. This design ensures that each sent request can still be matched to its corresponding response and checked even under asynchronous conditions, thereby achieving a 100% valid verification rate in our experiments.
- A concurrent testing architecture with optional LLM-assisted thread control. We implement asynchronous sender and verifier groups with bucket-locked access to the test object pool, together with an optional LLM-assisted thread controller that adapts the number of sending threads based on observed throughput. On an ICPS-like workload, the resulting framework improves effective verification throughput by up to 2.6 times compared with Apache JMeter, while maintaining the same or higher valid verification rate.

The remaining sections of this paper are organized as follows. Section 2 discusses related work. Section 3 describes the architecture of industrial cyber-physical systems, followed by Section 4, which presents the detailed algorithms designed for the implementation of the architecture. In Section 5 and Section 6, we evaluate the performance of the DIOTVF and conclude the paper.

## 2. Related Work

As industrial networked systems grow increasingly complex, ensuring the correctness, reliability, and scalability of testing methodologies has become a critical research challenge. This section reviews the relevant literature from three perspectives: distributed input–output testing, asynchronous concurrent testing, and LLM-assisted software testing [11]. The goal is to establish the necessary technical background and clarify how these areas of research motivate the DIOTVF proposed in this paper. These three perspectives directly correspond to the core technical challenges addressed in this paper: modelling and managing spatially distributed input/output paths, coping with random delays and out-of-order responses in concurrent testing, and exploring how large language models can help coordinate complex test tasks and resource scheduling.

### 2.1. Distributed Input–Output Testing

Research on distributed input–output testing has advanced rapidly alongside the growth of large-scale distributed services and industrial control systems. DistFuzz [12] introduces a feedback-guided blackbox fuzzing framework that treats requests, injected faults, and event timing as a multi-dimensional input space, using pruned network message sequences as feedback to uncover deep bugs. However, it still depends on carefully designed abstractions of events and messages, which are difficult to generalize across heterogeneous industrial protocols. Mallory [13] applies graybox fuzzing with multi-node execution feedback to perturb message sequences and fault injections, but its reliance on manual annotations and instrumentation increases adoption costs in production environments. MONARCH [14] proposes a scalable multi-node semantic coverage model for distributed file systems, enabling detection of cross-node inconsistencies, though its abstraction is tightly coupled to storage semantics and less suitable for general distributed I/O flows. DiamonT [15] enhances runtime observability by modelling alternative event orderings in asynchronous programs, offering strong diagnostic capabilities but not a systematic active testing workflow.

In distributed learning workloads, D3 [16] performs differential testing by generating model variants and inputs to surface inconsistencies across back-ends and configurations. While effective for deep-learning pipelines, it does not generalize to message-driven industrial protocols. Complementing fuzzing, Mahe et al. [17] present an interaction-based offline runtime verification approach using multi-trace lifeline removal to handle partial observations, which is well suited for log-based analysis but not for coverage-guided active testing of live systems. FieldFuzz [18] focuses on industrial automation runtimes by reverse-engineering the Codesys environment and combining network fuzzing with on-device tracing to uncover critical vulnerabilities. Although it demonstrates the practicality of blackbox distributed I/O fuzzing in industrial settings [4,19], it remains tightly tailored to the Codesys ecosystem and provides limited abstractions for orchestrating distributed I/O testing across diverse industrial networks. Beyond single-cluster fuzzing setups, Jang et al. [20] propose Fuzzing@Home, a distributed fuzzing framework that leverages untrusted heterogeneous clients at scale, while Chen et al. [21] redesign parallel fuzzing as a microservice-based architecture in µFUZZ to decouple coordination and execution; both works illustrate the scalability benefits of distributed test input generation, but they focus on general-purpose binaries rather than industrial protocols with spatially separated I/O. From the runtime-verification perspective, Audrito et al. [22] formalize distributed runtime verification using past-CTL and the field calculus, and Momtaz et al. [23] study monitoring of Signal Temporal Logic properties in distributed cyber-physical systems, showing how formal specifications can be evaluated across distributed inputs and outputs, although these efforts primarily target property checking rather than high-throughput active fuzzing under asynchronous network delays.

### 2.2. Asynchronous Concurrent Test

Asynchronous and concurrent behavior is a major source of complexity and flakiness in large-scale systems, motivating extensive work on specialized testing and runtime verification. Wolff et al. [24] apply graybox fuzzing to systematically explore thread interleavings with coverage-guided mutation and concurrency-aware feedback, exposing data races and atomicity violations. Although effective for shared-memory programs, it does not address long-latency, message-driven industrial workloads. Zhao et al. [25] propose selectively uniform concurrency testing (SURW), which explores representative equivalence classes of schedules to reduce redundant interleavings, but its abstractions remain tied to thread-level scheduling rather than distributed I/O tasks.

From a runtime verification viewpoint, Ang et al. [26] introduce predictive monitoring for pattern regular languages, enabling online detection of complex concurrent behaviors with controlled overhead. This provides expressive specifications but assumes integrated monitors and does not consider high-rate asynchronous test injection. Ganguly et al. [27] verify metric temporal properties under partial synchrony using bounded clock skew and SMT-based progression, showing how timing constraints can be enforced across nodes, yet the method targets runtime traces rather than orchestrated concurrent testing. Bonakdarpour et al. [28] develop decentralized, crash-resilient runtime verification that tolerates failures and delays, improving robustness in asynchronous settings, though the focus is on monitor reliability rather than scalable test generation. At the test-management level, Tahir et al. [29] provide a multivocal review of test flakiness, identifying concurrency-induced nondeterminism as a dominant cause and noting that industry still relies heavily on reruns instead of systematic modelling. Parry et al. [30] enhance rerun-based flaky-test detection using machine learning (CANNIER), yet this work continues to treat the executor as a black box without explicit control over asynchronous send/receive operations or large pools of concurrent test tasks. Therefore, recent research has started to direct concurrency fuzzing towards specific bug classes. Yuan et al. [31] present DDRace, a directed graybox fuzzer for discovering concurrency use-after-free vulnerabilities in Linux device drivers, Ito et al. [32] design Schfuzz to detect concurrency bugs via feedback-guided exploration of thread interleavings, and Ito et al. [33] further extend this line with race-directed fuzzing to focus testing effort on suspected race locations. Jiang et al. [34] complement these approaches with CONZZER, a context-sensitive and directional concurrency fuzzer specialized for device drivers, illustrating how schedule-sensitive exploration strategies can systematically expose concurrency defects but still operate at the program level rather than coordinating distributed I/O endpoints.

### 2.3. LLM-Assisted Software Testing

With the rapid advancement of large language models, extensive research now examines how LLMs can support or automate software testing. Wang et al. [35] survey over one hundred studies across testing tasks, model types, prompting strategies, and auxiliary techniques, highlighting persistent challenges in reliability, controllability, and security of LLM-generated tests. Li et al. [36] benchmark multiple LLMs on diverse testing tasks and show that, while LLMs can outperform traditional tools in certain cases, they still exhibit hallucinated assertions and unstable performance across projects, limiting their use in safety-critical contexts. Rehan et al. [37] propose an LLM-based pipeline that generates scalable test suites using prompt engineering and post-processing, demonstrating good results for conventional application code, though the approach remains focused on code-centric unit and functional testing rather than distributed or timing-sensitive interactions. Celik and Mahmoud [38] review LLM-driven test generation methods and identify research gaps such as tighter integration with runtime feedback and domain-specific models, noting that structured test representations could significantly improve LLM reliability.

Yuan et al. [39] present ChatTester, which iteratively refines LLM-generated unit tests to improve compilation and assertion correctness, but its design targets single-function testing and does not address concurrent or distributed behaviors. Li et al. [40] study LLM-generated web-form tests across 146 forms, showing that richer context and carefully crafted prompts substantially improve submission success rates, though the domain remains confined to web front ends. Expanding beyond task-specific work, Zhao et al. [41] introduce an AI-augmented QA framework that combines NLP-based requirement analysis, ML-based test generation and prioritization, and deep-learning-based anomaly detection within a PyTest-BDD workflow, illustrating how AI components can be integrated throughout a unified testing pipeline. Building on unit-level evaluations, Schäfer et al. [42] conduct a large-scale empirical study of LLM-based unit test generation for JavaScript libraries, while Dakhel et al. [43] introduce MuTAP, which augments LLM-generated tests with mutation testing to improve fault-detection effectiveness. Kang et al. [44] propose Libro, demonstrating that LLMs can act as few-shot testers that synthesize bug-reproducing tests directly from natural-language bug reports, and Meng et al. [45] integrate LLMs into protocol fuzzing by extracting protocol grammars and stateful message sequences for coverage-guided exploration of network services. Readers can refer to these works [46,47] for more discussion of the risks caused by LLM-driven methods.

Across the above lines of work, there is room for improvement in both specificity and universality. Approaches such as DistFuzz, Mallory, MONARCH, and FieldFuzz achieve strong bug-finding capability in their targeted ecosystems, but they are often tightly coupled to particular abstractions, protocol stacks, or runtime environments and may require substantial manual annotations or instrumentation effort to deploy in new industrial settings. On the other hand, log-based analysis and formal runtime verification frameworks (e.g., DiamonT, Mahe et al., Audrito et al., Momtaz et al.) offer powerful post-hoc guarantees, yet they do not provide a high-throughput, active testing workflow for live, delay-prone industrial networks. Our work complements these directions by focusing on a protocol-agnostic, template-driven active testing and verification framework that treats most of the system under test as a black box apart from configurable test and verification ports, explicitly models spatially distributed input/output paths via a shared test object pool, and scales to multithreaded crowdsourced execution without requiring special-purpose instrumentation of industrial controllers or industrial middleware.

## 3. The Design

### 3.1. System Architecture

**Figure 1:**
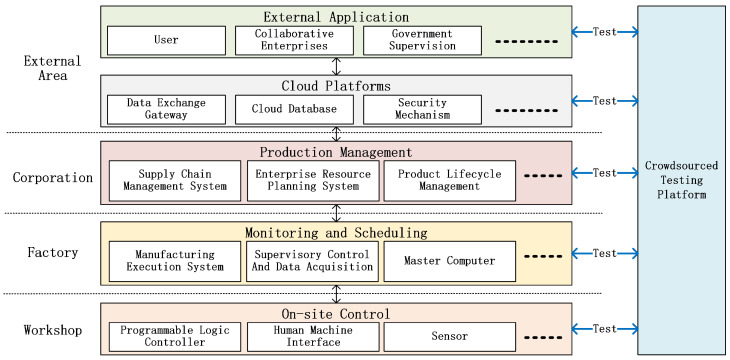
Test architecture of crowdsourced testing platform with industrial cyber-physical systems.

The test architecture of the crowdsourced testing platform is aligned with the layered structure of cloud-based industrial cyber-physical systems, which is shown in Figure 1. It consists of four interconnected layers: the workshop layer, factory layer, corporation layer, and external region layer. These layers reflect different enterprise functions and jointly integrate physical devices, software systems, network communication, and cloud services. Moreover, each layer interacts with the others to emulate realistic operational workflows: the workshop layer connects to physical equipment and collects data; the factory layer coordinates device operation and scheduling; the corporation layer manages resources across multiple factories; and the external region layer supports cross-enterprise data exchange through cloud platforms.

In a crowdsourced deployment, each workshop, factory, or external site can host one or more testing agents that register with the platform and periodically pull test objects from a shared test object pool. The proposed dynamic test–task management model and asynchronous concurrent testing model then execute these tasks across all agents, while the central platform aggregates testing and verification results for further analysis. In other words, the crowdsourced platform provides global coordination, task distribution, and result collection, whereas the DIOTVF described below specifies the concrete mechanisms used by agents and the central service to generate, send, match, and verify test payloads.

**Figure 2:**
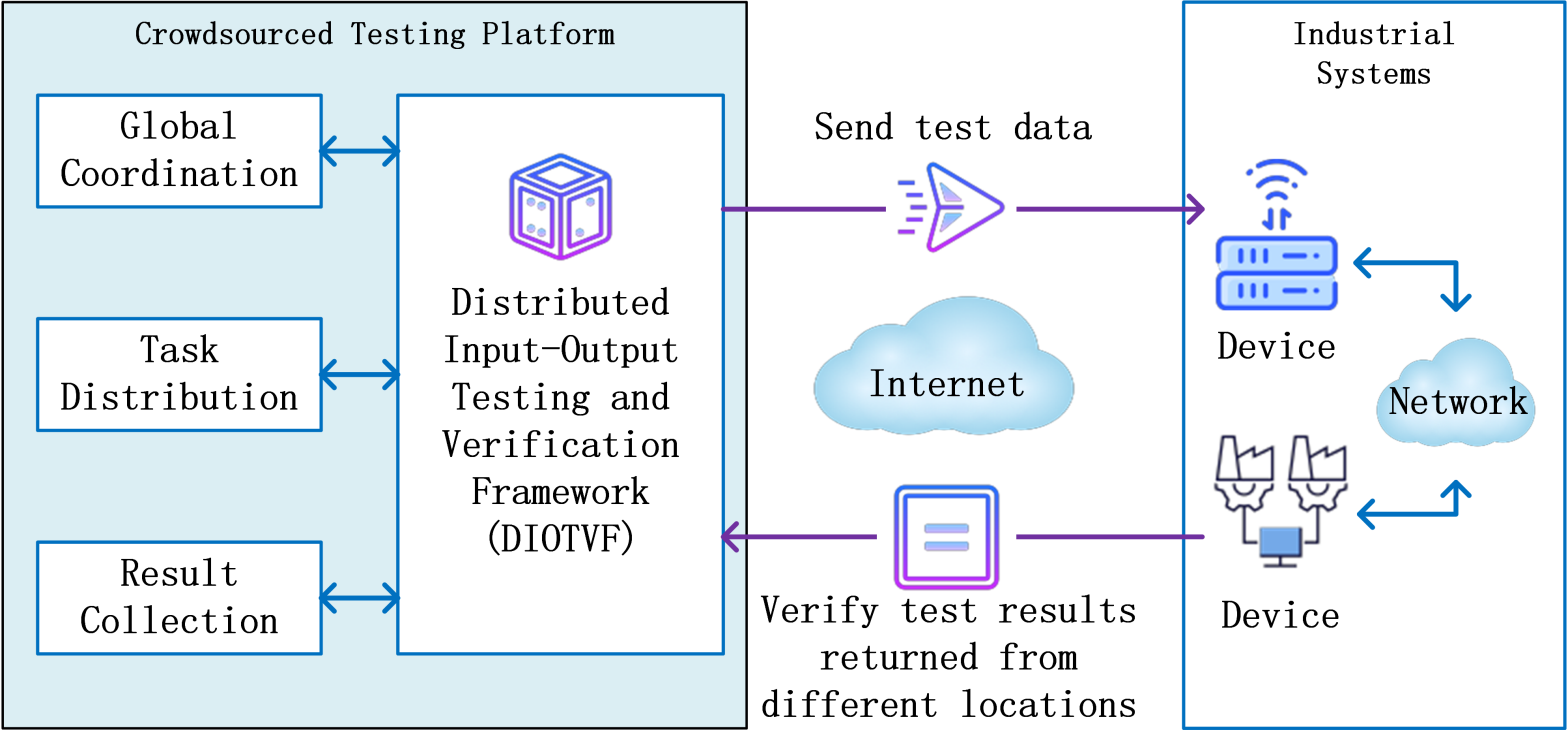
Test architecture of crowdsourced testing platform with distributed input–output testing and verification framework.

From the perspective of the crowdsourced testing platform, industrial systems often exhibit mismatches between the locations where input data are injected and where output data are collected, due to the distributed and complex nature of their operational processes. To address the testing challenges arising from this input–output asymmetry, we propose a distributed input–output testing and verification framework, illustrated in Figure 2. In this architecture, the system under test is assumed to have geographically or logically separated input and output points, with internal devices interconnected through the industrial network to form a complete data-flow path. During testing, the framework sends test payloads over the Internet to designated input-side devices within the industrial system, while continuously retrieving output data from devices located elsewhere in the network. After collecting the output, the testing tool validates the returned data against the original inputs and performs statistical analysis to evaluate correctness and performance. To support this process, we introduce two key models: a dynamic test–task management model and an asynchronous concurrent testing model. Together, these models enable reliable dispatching of test data and accurate verification of results across distributed locations, ensuring that input–output testing remains effective in complex, heterogeneous industrial environments.

### 3.2. Dynamic Test–Task Management Model

**Figure 3:**
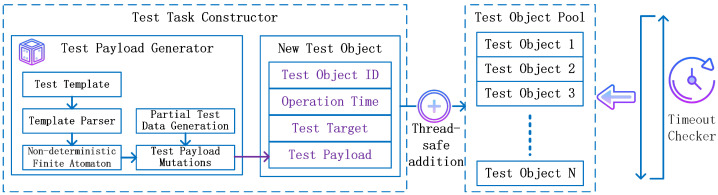
Dynamic test–task management model.

To effectively manage distributed testing tasks, this paper proposes a dynamic test–task management model, which is used to implement test payload generation, the addition of test–task objects, and the dynamic management of the test object pool. The model’s workflows are shown in Figure 3. The dynamic test–task management model consists of three components: the test–task constructor, the test object pool, and the timeout checker. By integrating these three functions within the dynamic test–task management model, the entire execution of the testing tasks can be effectively supported.

#### 3.2.1. Test Payload Generator

The test–task constructor includes several key functions: data parsing for filling the test template, generation of partial test payloads for test mutations, merging mutated payloads into new test objects, and setting the basic attributes of the new test objects, such as the test targets, identifiers, and the last operation time. To support the proper functioning of the test–task constructor, this paper defines the test template, denoted as tmpl, which can be expressed as Equation (1).

(1) $$tmpl=\{headtmpl,\sum_{i=1}^{n}\left(rep_{i},repfill_{i}\right),reartmpl\}$$

where headtmpl represents the header data of the template, with a length greater than or equal to 0; reartmpl represents the rear data of the template, with a length greater than or equal to 0; $rep_{i}$ denotes the data in the template that needs to be mutated and replaced, and $repfill_{i}$ represents the payload data after the mutation replacement in the template. To ensure the proper operation of the template replacement mechanism, *i* should be at least 1, and $repfill_{i}$ should be greater than or equal to 0. Additionally, for effective marker replacement, $rep_{i}$ should be longer than the replacement marker length, and $rep_{i}$ can be expressed as Equation (2).

(2) $$rep_{i}=\left\{repMark,repID,repLen,repMark\right\}$$

where repMark represents the identifier region to be replaced, used to mark the start and end; repID is the identifier for the replacement region; and repLen is the length of the data to be generated for the replacement.

After the test user completes the test template creation, the test payload constructor needs to parse the information marked by $rep_{i}$. At this point, this paper employs a non-deterministic finite automaton (NFA) approach for template matching and positioning. This model efficiently identifies and replaces the necessary parts of the template, ensuring that dynamically mutated test payloads are correctly processed during the testing process. For a non-deterministic finite automaton, it is typically composed of the five-tuple as shown in Equation (3).

(3) $$A=(Q,\Sigma ,\delta ,q_{0},F)$$

where *Q* is the set of states (including states for matching headtmpl, $rep_{i}$, and reartmpl), Σ is the input alphabet (the set of possible characters or symbols in the template), δ is the transition function, which defines how the NFA moves between states based on the input symbols, $q_{0}$ is the initial state, where the matching begins (specifically matching headtmpl), *F* is the set of final states, indicating a successful match of the entire template. Thus, the matching process can be formally expressed as Equation (4).

(4) Match(tmpl)=NFA(repMark,repID,repLen,repMark)

In this expression, the process first matches repMark, followed by repID and repLen, and finally matches repMark again. During this matching process, the NFA transitions between different states, ultimately reaching a final state when the match is successful. Once the NFA successfully identifies the entire template in the input data, the next step is to perform the replacement. At the same time, before performing the template mutation payload replacement, the test data to be replaced must first be generated. During the generation process, the replacement data, denoted as td, must satisfy Equation (5).

(5) td=Gen(repLen)

where Gen represents the length of data to be generated. In practical use, the data will be generated according to the repLen length extracted from the NFA matching and recognition results, ensuring the generation of the corresponding test data. After generating td, it needs to be replaced in the corresponding position of tmpl, resulting in the mutated test payload, denoted as pl, which must satisfy Equation (6).

(6) $$pl=\{headtmpl,\sum_{i=1}^{n}\left(td_{i},repfill_{i}\right),reartmpl\}$$

where headtmpl represents the header data of the template; reartmpl represents the rear data of the template; $repfill_{i}$ is the original padding data for the corresponding replacement section. Meanwhile, $td_{i}$ is the newly generated mutated replacement data.

#### 3.2.2. New Test Object

After generating the test payload, a test object needs to be constructed to support the execution of the testing tasks. The test object is denoted as to and should satisfy Equation (7).

(7) to={toid,time,target,pl}

where toid is the identifier of the test object, time represents the last operation time of the test object, target refers to the test target defined for the test object, and pl is the test payload. The identifier of the test object, toid, must satisfy Equation (8).

(8) toid=ltoid+1

where ltoid represents the ID of the last constructed test object, which should be of a numeric type that supports atomic operations to avoid potential issues where the same toid is generated during concurrent multithreaded operations. Additionally, the test target defined for the test object, target, must satisfy Equation (9).

(9) target={testTarget,verTarget}

where testTarget refers to the information about the location where the test payload needs to be sent, while verTarget refers to the test location information for retrieving the test results.

#### 3.2.3. Test Object Pool Addition

In the dynamic management of the test object pool, the test object pool contains a set of test objects that are currently being processed. Therefore, in this paper, the test object pool is denoted as top, which consists of a set of test objects to, and can be expressed as Equation (10).

(10) $$top=\{to_{1},to_{2},\ldots ,to_{n}\}$$

where *n* represents the number of test objects currently present in the test object pool. At the same time, since the test object pool can be operated by multiple threads concurrently, it must support thread-safe operations. To improve the performance of multithreaded operations, and given that the test object identifier is used as the primary key, the model is implemented using a basic bucket lock scheme. In this case, the number of bucket locks can be defined as Equation (11).

(11) $$bucket=diff(\left\{H\left(to_{1}\right),H\left(to_{2}\right),\ldots ,H\left(to_{n}\right)\right\})$$

where *H* is the hash function used to obtain the hash value of the currently operated object; diff is used to merge identical elements within the set. Therefore, the total number of buckets in the collection is always less than or equal to the number of elements in the test object pool. Once the bucket locks are constructed, each thread-safe addition operation requires locking the corresponding bucket, and the lock is released after the addition is complete. For read operations, however, a lock-free approach is used.

#### 3.2.4. Remove Expired Test Objects

In the process of removing expired test objects, the timeout checker dynamically checks the elements in the test object pool to remove test objects that have not returned matched test data after a prolonged period since they were sent. By periodically removing expired test objects, the storage pressure on the test object pool is reduced, and the retrieval efficiency of the pool is improved. After the timeout checker completes the check, expired test objects are removed based on the time difference. The removed test object, denoted as RTO, is defined as Equation (12).

(12) $$RTO=\{rto_{1},rto_{2},\ldots ,rto_{n}\}$$

where each rto∈RTO satisfies st−rto.time>timeoutSpan; all removed expired test objects must have elapsed more than the defined timeout period since the current check time; and *n* is the number of expired test objects that were removed.

### 3.3. Asynchronous Concurrent Testing Model

**Figure 4:**
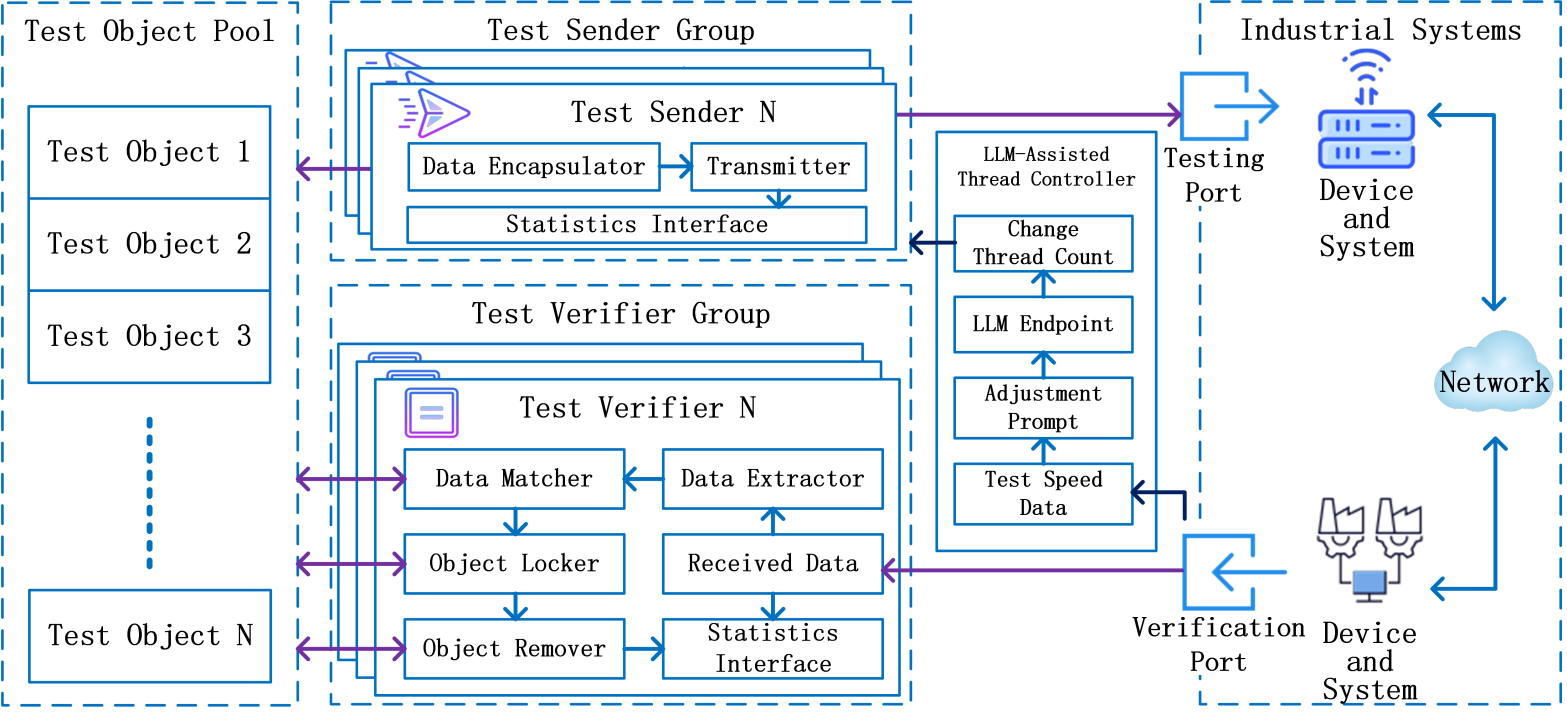
Asynchronous concurrent testing model.

After constructing the test tasks and adding them to the test object pool, the DIOTVF needs to implement the specific testing tasks using asynchronous concurrent testing. Therefore, this paper proposes the asynchronous concurrent testing model, with the overall working principle illustrated in Figure 4. In the asynchronous concurrent testing model, based on the test object pool provided by the dynamic test–task management model, a test sender group, a test verifier group, and a large language model-assisted (LLM-assisted) thread controller are introduced [46]. By integrating these key components, the model can effectively perform testing on distributed input–output objects.

#### 3.3.1. Test Sender Group

The test sender group is responsible for concurrently executing test tasks according to the testing configurations defined within the test object pool. In this paper, the test sender group is denoted as tsg, which consists of a set of test senders, defined as Equation (13).

(13) $$tsg=\{ts_{1},ts_{2},\ldots ,ts_{n}\}$$

where $ts_{i}$ represents a single test sender, and *n* denotes the total number of test senders, which also corresponds to the number of sending threads in the implementation. After the test sender group is initialized, each test sender extracts a test object from the test object pool to initiate its testing operation. Since the sending process is performed asynchronously and concurrently, the test senders do not follow a fixed order when accessing test objects. To describe this non-sequential access relationship, a mapping is defined as Equation (14).

(14) f:tsg→top

where $f\left(ts_{i}\right)=to_{j}$ indicates that the test sender $ts_{i}$ retrieves the test object $to_{j}$ from the test object pool for testing. This mapping satisfies the uniqueness constraint shown in Equation (15).

(15) $$\forall i\neq k,\;f\left(ts_{i}\right)\neq f\left(ts_{k}\right)$$

which ensures that no two test senders retrieve the same test object. In addition, the access order of test objects is not constrained and can be expressed as Equation (16).

(16) $$f\left(ts_{i}\right)=to_{\pi (i)},\;\pi \in S_{m}$$

where π is a permutation function on {1,2,…,m} representing the non-sequential assignment of test objects. Therefore, the access behavior of the test sender group exhibits both non-sequentiality and uniqueness. After completing the extraction of test objects, each test sender reads the corresponding test payload and configures the transmission target according to the defined testing objectives. Let the payload of the test object $to_{j}$ be denoted as $pl_{j}$, and its transmission target as $target_{j}$. Then, the transmission target configuration expression is shown as Equation (17).

(17) $$g\left(ts_{i}\right)=pl_{j},\;tgt\left(ts_{i}\right)=target_{j},\;\mathrm{where}\;f\left(ts_{i}\right)=to_{j}$$

Once the payload retrieval and target configuration are completed, the test sender performs the sending operation and collects statistical information regarding the transmission. Let σ(·) denote the sending statistics function, which records parameters such as transmission time, data size, and send count. The sending statistics can then be expressed as Equation (18).

(18) $$stat\left(ts_{i}\right)=\sigma \left(pl_{j},target_{j}\right)$$

Hence, based on the non-sequential and unique extraction of test objects, each test sender accomplishes a complete operational process, including payload reading, target configuration, and data transmission statistics, providing quantitative support for subsequent verification and performance analysis.

#### 3.3.2. Test Verifier Group

The test verifier group is responsible for receiving, matching, and validating the response data generated by the test sender group, thereby determining the execution results of the test tasks. In this paper, the test verifier group is denoted as tvg, which consists of a set of test verifiers defined as Equation (19).

(19) $$tvg=\{tv_{1},tv_{2},\ldots ,tv_{n}\}$$

where $tv_{i}$ represents an individual test verifier, and *n* denotes the number of verifiers, corresponding to the number of concurrent verification threads in the system. Once the test verifier group is activated, each test verifier receives response data from the system under test through the verification port and matches it with the corresponding test objects stored in the test object pool. Let the set of received response data be represented as $rd=\{rd_{1},rd_{2},\ldots ,rd_{m}\}$, then the verification mapping can be defined as Equation (20).

(20) h:tvg→rd

where $h\left(tv_{i}\right)=rd_{j}$ indicates that the test verifier $tv_{i}$ is responsible for validating the response data $rd_{j}$. To correctly associate each received response with its corresponding test object, a data matching function is defined as Equation (21).

(21) $$match\left(rd_{j}\right)=to_{k},\;\mathrm{where}\;h\left(tv_{i}\right)=to_{k}$$

This means that the verifier establishes the correspondence between the received response data and its associated test object through the matching function to complete the validation task. After successful matching, the verifier performs object locking and removal operations to prevent duplicate verification. Let the locking and removal operations be denoted by lock(·) and remove(·), respectively, as shown in Equation (22).

(22) $$lock\left(to_{k}\right),\;remove\left(to_{k}\right)\in top$$

where lock(·) marks the test object currently under verification, and remove(·) indicates that the verified test object has been removed from the test object pool after the verification process is completed. Finally, to quantitatively analyze the verification performance, let ρ(·) denote the verification statistics function, which records metrics such as verification count, matching success rate, and data loss rate. The statistical relation can thus be expressed as Equation (23).

(23) $$stat\left(tv_{i}\right)=\rho \left(rd_{j},to_{k}\right)$$

Accordingly, the test verifier group completes the entire verification workflow—including response reception, data matching, object locking, and statistical analysis—thereby enabling asynchronous validation and performance evaluation of test–task execution results.

#### 3.3.3. LLM-Assisted Thread Controller

The LLM-assisted thread controller is designed as an optional auxiliary module that dynamically adjusts the number of concurrent threads in the test sender group based on real-time performance data collected during the testing process. Its primary objective is to balance system load and testing throughput while maintaining test accuracy, thereby improving overall efficiency and resource utilization. In the asynchronous concurrent testing model, the execution speeds of the test sender group and the test verifier group may differ due to factors such as network latency, device response time, and system resource consumption. To address these variations, an LLM-based thread control mechanism is introduced to perform adaptive analysis and adjustment of the system state. Similar to Deng et al.’s work [48], we introduce a data selection technique to make sure the LLM-based mechanism is trustworthy. Let the test speed data set be denoted as $spd=\{s_{1},s_{2},\ldots ,s_{t}\}$, and the thread control function be denoted as θ(·). The mapping relationship for thread adjustment can be defined as Equation (24).

(24) $$\theta :spd\to n^{\prime }$$

where $n^{\prime }$ represents the adjusted number of testing threads, and the adjustment strategy of θ(·) is inferred by the LLM based on performance prompts provided as input. The controller first collects statistical data such as testing throughput and resource utilization, then converts these indicators into prompt information and submits them to the LLM endpoint. The LLM analyzes the current system load and performance state and generates thread adjustment recommendations. The controller then updates the number of threads in the test sender group according to these recommendations, achieving adaptive scheduling of testing workloads. Let φ(·) denote the thread adjustment recommendation function generated by the LLM. The final adjustment relationship can be expressed as Equation (25).

(25) $$n^{\prime }=\varphi \left(\theta \left(spd\right)\right)$$

Through this mechanism, the system can automatically optimize the concurrency scale based on model inference results under scenarios with varying testing speeds or complex resource competition, thereby enabling dynamic self-scheduling and intelligent optimization of testing tasks. It should be noted that the LLM-assisted thread controller is an optional component and may be disabled in environments with sufficient resources or small-scale testing tasks to reduce computational overhead.

## 4. The Implementation

To realize the practical implementation of the DIOTVF described in the previous section, this paper proposes a systematic method that translates the conceptual models into executable algorithmic procedures. The proposed method focuses on enabling automated, scalable, and concurrent testing of industrial systems with distributed data input and output positions. It integrates the dynamic test–task management model and the asynchronous concurrent testing model into a cohesive operational workflow, covering test payload generation, concurrent sending, timeout management, and asynchronous verification. Each algorithm is designed to handle a specific stage of the distributed testing process, while collectively ensuring correctness, efficiency, and adaptability. The overall methodology aims to support large-scale industrial software testing where spatially separated devices and asynchronous data flows can be tested under realistic network conditions. The detailed algorithmic procedures are presented as follows.

### 4.1. Test Payload Generation

To enable large-scale and automated testing of distributed input–output systems, this paper first proposes a template-driven test payload generation algorithm, which is shown in Algorithm 1. The approach employs a non-deterministic finite automaton (NFA) to locate and replace variable regions within the test template, ensuring that the generated payloads conform to the expected structural and semantic rules of the target industrial protocol. Through dynamic filling and mutation of template parameters, diversified test payloads are generated to comprehensively stimulate different states of the system under test. After each payload is constructed, the test–task constructor encapsulates it into a test object, assigns a unique identifier, and inserts it into the test object pool in a thread-safe manner for subsequent concurrent testing.
**Algorithm 1** Test payload generating**Input:** Test template tmpl**Output:** Test payload pl and constructed test object to 1:Initialize non-deterministic finite automaton $A=(Q,\Sigma ,\delta ,q_{0},F)$ for template parsing 2:**for** each replacement region $rep_{i}$ in tmpl **do** 3:    Locate repMark,repID,repLen via A transitions 4:    Generate replacement data $td_{i}=Gen\left(repLen\right)$ 5:    Substitute $rep_{i}$ with $td_{i}$ and append padding $repfill_{i}$ 6:Assemble final payload pl 7:Construct new test object to with atomic toid increment 8:Add to into test object pool top through thread-safe addition 9:**return** to

### 4.2. Concurrent Test Payload Sending

After the test payloads are generated and stored in the test object pool, the system proceeds to concurrent test execution. The concurrent test payload sending algorithm is illustrated in Algorithm 2. The proposed asynchronous concurrent testing model enables multiple sender threads to operate in parallel, where each test sender retrieves a unique test object from the pool without a fixed access order. This non-sequential and thread-safe mechanism ensures that no two senders process the same object simultaneously. Each sender reads the corresponding payload and target configuration, performs transmission through the testing port, and logs transmission statistics for further analysis.

**Figure 5:**
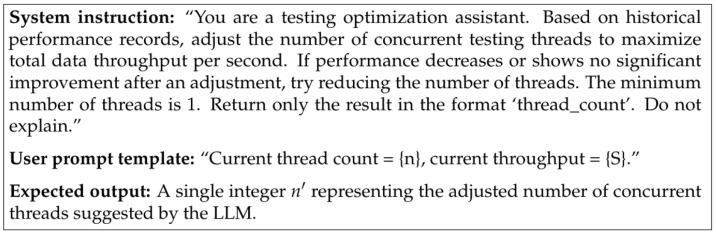
Prompt Template Used in the LLM-assisted Thread Controller.

To maintain an optimal balance between throughput and system load, an optional LLM-assisted thread controller is employed. Instead of relying on fixed hand-crafted rules, the controller uses the current throughput and thread count as feedback to decide whether the number of sending threads should be increased, decreased, or kept unchanged. The prompt template used in the LLM-assisted thread controller is shown in Figure 5. In practice, the sending procedure is organized into consecutive time windows. At the end of each window, the framework aggregates the transmission statistics from all sender threads, computes the current throughput *S* for that window, and invokes the LLM controller once. All invocations are issued within the same LLM session so that the model can see the sequence of previous “Current thread count = {n}, current throughput = {S}.” prompts as the historical performance records mentioned in the system instruction. For each window, the controller forms a short user prompt that encodes the current thread count and the measured throughput, sends it together with the fixed system instruction, and receives a recommended new thread count for the next window. Before applying this recommendation, the framework enforces several simple safety rules: the new thread count must stay within a configured minimum and maximum range; the difference between the previous and the new value is limited to a small step size to avoid oscillation. To avoid blocking normal packet transmission, the LLM controller is only triggered after a window completes, and the framework degrades gracefully to a fixed-thread mode when the LLM endpoint is unavailable.
**Algorithm 2** Concurrent test payload sending**Input:** Test object pool $top=\{to_{1},to_{2},\ldots ,to_{m}\}$, sender group $tsg=\{ts_{1},ts_{2},\ldots ,ts_{n}\}$**Output:** Transmission statistics set $\sigma =\{\sigma_{1},\sigma_{2},\ldots ,\sigma_{n}\}$ 1:**for all** sender $ts_{i}$ in tsg **in parallel do** 2:    Randomly select $to_{j}$ from top where $f\left(ts_{i}\right)=to_{j}$ and $to_{j}$ not locked 3:    Extract payload $pl_{j}$ and transmission target $target_{j}$ 4:    Perform transmission $\mathrm{Transmit}(pl_{j},target_{j})$ via transmitter 5:    Record send statistics $\sigma_{i}=\sigma \left(pl_{j},target_{j}\right)$ 6:Aggregate per-sender statistics over the current time window to compute the current throughput *S* 7:**if** LLM controller is enabled and the adjustment condition is satisfied **then** 8:    Build the user prompt: “Current thread count = {n}, current throughput = {S}.” 9:    Send the fixed system instruction and the user prompt to the LLM service10:   Receive the recommended thread count $n^{\prime }$11:   Clamp $n^{\prime }$ into the configured minimum and maximum range12:   Limit the difference between $n^{\prime }$ and *n* to a small step size13:   Resize sender group tsg to use $n^{\prime }$ threads in the next window14:   Update $n\leftarrow n^{\prime }$15:**return** σ

### 4.3. Timeout Test Payload Management

During large-scale testing, some payloads may remain unmatched for extended periods due to network latency, device response failures, or transmission errors. Therefore, timeout test payload management is necessary, and the algorithm shown in Algorithm 3. To prevent memory overflow and reduce retrieval overhead, a timeout checker continuously monitors the test object pool. If the elapsed time since an object’s last operation exceeds the predefined timeout threshold, that object is marked as expired and safely removed. This mechanism ensures efficient memory utilization and maintains high system throughput under long-duration distributed testing conditions.
**Algorithm 3** Timeout test payload management**Input:** Test object pool top, system time st, timeout threshold timeoutSpan**Output:** Removed expired test objects $RTO=\{rto_{1},rto_{2},\ldots ,rto_{k}\}$1:Initialize RTO←∅2:**for** each $to_{i}$ in top **do**3:    **if** $st-to_{i}.time>timeoutSpan$ **then**4:        Lock $to_{i}$5:        Remove $to_{i}$ from top6:        Append $to_{i}$ to RTO7:Update pool metrics to maintain load balance8:**return** RTO

### 4.4. Concurrent Retrieval and Asynchronous Analysis of Test Results

Once the test payloads have been transmitted, the verification layer asynchronously collects response data returned from different locations of the industrial system. Therefore, concurrent retrieval and asynchronous analysis of the test results are required, with the algorithm shown in Algorithm 4. Each test verifier thread retrieves one piece of response data, performs matching with the corresponding test object, and verifies whether the returned data satisfies the expected behavior. Upon successful validation, the verifier locks and removes the associated object from the pool to avoid duplicate matching and then updates the verification statistics. This asynchronous design enables the real-time evaluation of distributed test results while maintaining high throughput and low coupling between sending and verification threads. Aggregated performance indicators—such as matching success rate, latency, and data loss ratio—are computed to provide quantitative metrics for subsequent performance analysis.
**Algorithm 4** Concurrent retrieval and asynchronous analysis of test results**Input:** Response data set $rd=\{rd_{1},rd_{2},\ldots ,rd_{m}\}$, verifier group $tvg=\{tv_{1},tv_{2},\ldots ,tv_{n}\}$, test object pool top**Output:** Verification statistics set $\rho =\{\rho_{1},\rho_{2},\ldots ,\rho_{n}\}$ 1:**for all** verifier $tv_{i}$ in tvg **in parallel do** 2:    Receive response $rd_{j}$ from verification port 3:    Find matched object $to_{k}$ using $match\left(rd_{j}\right)=to_{k}$ 4:    **if** match succeeds **then** 5:        $lock(to_{k})$; perform validation $\mathrm{Verify}(rd_{j},to_{k})$ 6:        $remove(to_{k})$ from top 7:        Record statistics $\rho_{i}=\rho \left(rd_{j},to_{k}\right)$ 8:    **else** 9:        Increment mismatch or loss counter10:Compute aggregated verification metrics (success rate, latency, data loss)11:**return** ρ

## 5. Evaluation

In this section, we evaluate the proposed DIOTVF from three perspectives: data exchange speed during testing and verification, the number of successful verifications, and the overall valid verification rate. Moreover, valid verification rate refers to a low-level, per-request metric: the fraction of test objects in the test object pool whose responses can be correctly matched and checked against their originating payloads under asynchronous delays, i.e., a 100% valid verification rate means that every test request issued during an experiment was successfully matched with a corresponding response and subjected to the expected output check. We do not claim that the test suite covers all functional requirements or execution paths of the system under test; coverage and requirement engineering are orthogonal to the framework proposed in this paper.

### 5.1. System Configuration

Multiple virtual machines were deployed on the virtualization platform for the experiment, each configured with 8 Intel(R) Xeon(R) Gold 6128 with 3.40 GHz vCPUs and 16 GB of memory. To simulate cross-network communication, random transmission delays were injected between the testing and verification interfaces. Accordingly, the experiments included both zero-delay tests and tests with randomly added delays ranging from 1 ms to 10 ms. Performance was measured under thread counts of 2 and 4. The proposed method requires both a sender and a receiver to operate concurrently, with each running in its own thread. As a result, the total number of threads must be even, making single-threaded testing infeasible; therefore, no single-thread results are reported for our method. For comparison, we selected three additional tools: Apache JMeter, Locust, and a baseline implementation of our approach written in the same programming language to emulate JMeter (denoted as baseline). Unlike our framework, these control-group tools execute a send-then-receive sequence in a single thread, allowing single-thread tests. To fairly evaluate sequential execution behavior, the test target was configured to return exactly one test payload requiring verification per request.

### 5.2. Evaluation of Valid Testing and Verification Rate

The main contribution of this paper is to propose a DIOTVF that supports distributed unpredictable delays. To effectively assess the success rate of testing under distributed unpredictable delays, we evaluated the valid testing verification rate, and the results are reported in Table 1. The results show that under conditions of multithreaded contention and varying delays, the proposed method, by utilizing the test object pool, is able to search for and verify previously sent test payloads, even in the case of unordered responses due to unpredictable delays, thereby ensuring that every test payload sent in the experiment can still be matched with its corresponding response and checked, even under random delays and multithreaded contention. Meanwhile, with one thread and no unpredictable delays, other methods also managed to achieve 100% verification due to sequential execution. However, with thread counts greater than one, random competition and querying resulted in valid verification rates as low as 54% (e.g., when four threads were tested simultaneously without unpredictable delays). Additionally, under conditions of unpredictable delay, the valid verification rates of both Apache JMeter and the baseline method (which was implemented following JMeter principles) do not apply because their test–task scheduling mechanisms are designed to verify the test payload as soon as it is sent, resulting in JMeter and the baseline approach almost always having each thread verify a test payload sent by another thread earlier in the test. Locust, due to its sending mechanism, achieved a verification rate of approximately 66.67%. Overall, these results indicate that the proposed framework can maintain a consistently high valid verification rate even in the presence of asynchronous delays, which is essential for trustworthy testing of ICPS-like workloads.

### 5.3. Evaluation of Successful Verification Count

**Figure 6:**
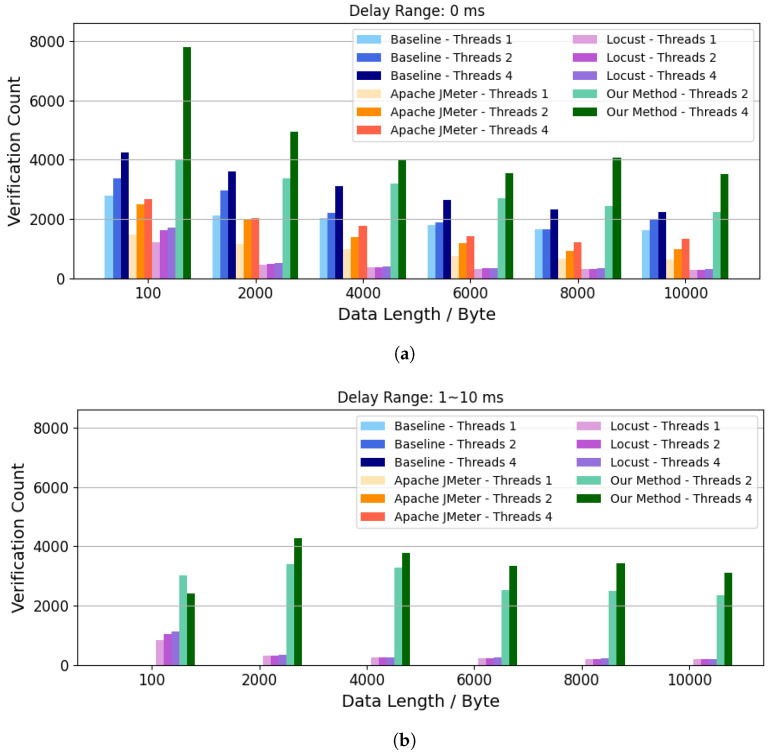
Comparison of successful verification count. (**a**) Data Return Delay Range = 0 ms; (**b**) Data Return Delay Range = 1 ∼ 10 ms.

The experimental results for the successful verification count are shown in Figure 6. The results show that the proposed method outperforms all the competitors in tests with different delay ranges. Specifically, with no additional unpredictable delays, the proposed method achieved a 0.87 times increase in verification count compared to the baseline group when four threads are used, and the data length is 8000 bytes. It achieved a 2.6 times increase compared to Apache JMeter and a 12.24 times increase compared to Locust. Under conditions of unpredictable delay, both JMeter and the baseline method produced zero valid verifications under our definition, and their valid verification rate is therefore considered not applicable in this delayed setting. Compared to Locust, which had a non-zero verification rate, the proposed method achieved a 15.63 times increase in verification count at the test payload size of 10,000 bytes. These observations confirm that the DIOTVF can sustain a high volume of correctly matched verifications in the presence of concurrent traffic and non-deterministic network delays.

### 5.4. Evaluation of Data Verification Speed

**Figure 7:**
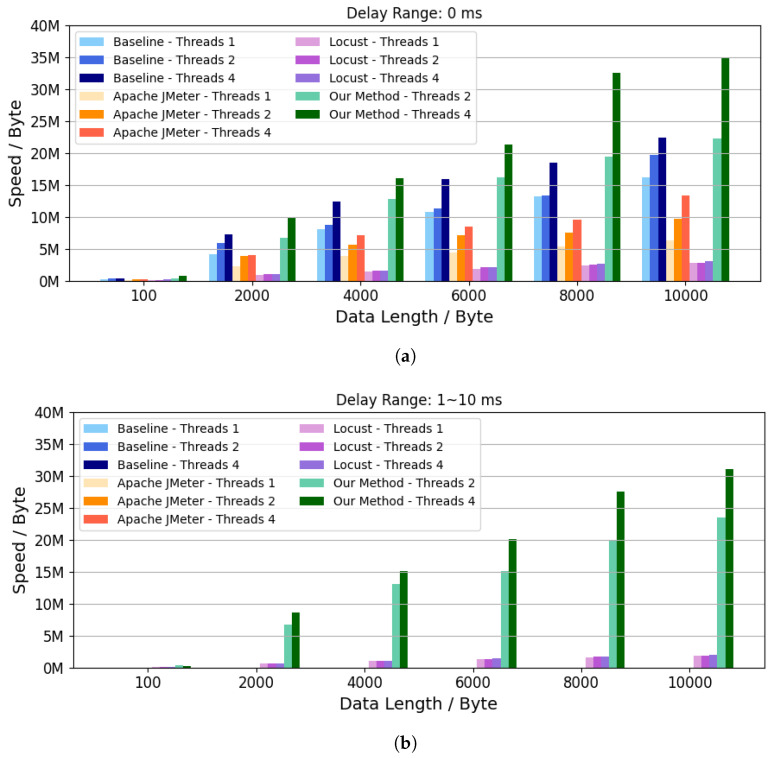
Comparison of data verification speed. (**a**) Data Return Delay Range = 0 ms; (**b**) Data Return Delay Range = 1 ∼ 10 ms.

The experimental results of data exchange speed are shown in Figure 7. As can be seen, the proposed method yielded higher data verification speeds with two threads compared to the other methods, even when they used four threads, regardless of the delay range. Since sending speed and data packet size are proportionally related, performance improvement rates are consistent with successful verification counts. With four threads and a 10,000-byte payload, the proposed method achieved an additional 23.53 MB/s in data verification speed compared to JMeter without unpredictable delays, and an additional 30.53 MB/s in data verification speed compared to Locust with unpredictable delays. In summary, the proposed framework not only improves the number of successful verifications but also significantly increases effective throughput, which is crucial for large-scale ICPS testing campaigns.

### 5.5. Discussion of ICPS-Specific Metrics

Although the proposed experiments are conducted on an ICPS-like protocol, the evaluation configuration is designed to approximate several key ICPS characteristics while maintaining accuracy and reproducibility. First, the injected delay range of 1–10 ms reflects typical network latency and jitter observed in many industrial deployments, where control messages are expected to complete within tens of milliseconds. Under these conditions, the framework maintains a 100% valid verification rate, suggesting that it can preserve correct input–output correspondence without violating soft real-time constraints in the tested regime. Each request/response pair emulates a control-cycle update, so the successful verification count and valid verification rate together indicate how many control cycles can be reliably exercised and checked under concurrent test traffic. By separating senders and verifiers and explicitly modelling out-of-order responses, the framework can also respect protocol-level requirements such as strict ordering between logically related messages, even when physical delivery order is perturbed by delays.

From the viewpoint of experimental accuracy and reproducibility, the explicit modelling of test objects and their life cycle further helps to tame nondeterminism in distributed experiments. Because each request is tagged with a unique identifier and can only be matched once by the verifier group, the framework enforces a one-to-one correspondence between sent payloads and verified responses even under asynchronous delays and multithreaded contention. For a fixed configuration (number of threads, delay distribution, and payload templates), the set of successfully verified test objects is therefore determined by the pseudo-random generator used to construct payloads, which makes comparative experiments repeatable on the same testbed. At the same time, we acknowledge that our current evaluation is limited to a single virtualized environment. A more fine-grained analysis involving hardware-in-the-loop PLCs, detailed scan-cycle measurements, and protocol-specific timing profiles, as well as extending the study to larger heterogeneous deployments and long-running crowdsourced campaigns where node churn and scheduler interference are more pronounced, remains important future work for fully characterizing ICPS relevance and reproducibility at scale.

### 5.6. Scalability and Complexity Discussion

From an algorithmic perspective, the main overhead of the proposed framework comes from maintaining the test object pool, matching responses to test objects, and parsing and mutating protocol payload templates. Let *N* denote the number of active test objects in the pool. The pool is implemented as a hash-based data structure with bucket locks, so insertion, lookup, and removal of test objects are O(1) on average. Response matching is also O(1) with respect to *N*, because each response carries an identifier that is used as a hash key to locate its corresponding test object.

Moreover, for the ICPS protocol used in our evaluation, this cost is dominated by network latency and the processing time of the system under test. As the size of the ICPS and the complexity of its protocols grow, the dominant factor in overall runtime is therefore the volume of test traffic and the number of active test objects, rather than the asymptotic complexity of our framework.

At the same time, the memory footprint of the test object pool grows linearly with the number of in-flight test objects. Very large-scale ICPS deployments with thousands of concurrent test threads or extremely large payloads would require careful resource provisioning. A more detailed scalability study that covers heterogeneous industrial protocols, deeper protocol stacks, and multi-site deployments is left as future work.

## 6. Conclusions

In summary, this work presents a DIOTVF within a distributed crowdsourced platform for ICPS security testing and makes three main contributions. The crowdsourced platform provides the architectural context in which heterogeneous testing agents are coordinated, while the focus of this paper is on the design, implementation, and evaluation of the distributed input–output framework that serves as the platform’s core testing engine. Built on a four-layer cloud-based ICPS architecture, the proposed distributed crowdsourced testing architecture decouples heterogeneous testing agents from the system under test via a shared test object model and standardized test/verification ports, thereby supporting proactive, cross-network, cross-device, and cross-layer security testing under realistic deployment conditions. On top of this architecture, we design a delay-aware input–output testing and verification framework that uses a hash-based test object pool and explicit timeout management to maintain correct request–response matching under unpredictable communication delays, and combine a dynamic test–task management model with an asynchronous multithreaded testing architecture, optionally enhanced by an LLM-assisted thread controller, to support complete test verifications even in distributed environments with delays induced by data processing or network instability between test and verification ports. Experimental results show that, compared to Apache JMeter, Locust, and a baseline implementation, the proposed framework can achieve up to 2.6 times improvement in verification efficiency while maintaining a 100% valid verification rate in the sense defined in Section 5, i.e., every test request is matched to a response and successfully checked.

In future work, we plan to extend the proposed framework along three directions. First, we will further enrich ICPS-specific evaluation, including tighter integration with hardware-in-the-loop PLCs, scan-cycle and jitter measurements, and protocol-aware behavioral metrics. Second, we aim to explore dynamic test templates and adaptive construction of test payloads based on intermediate verification results, in order to support fuzzing-style exploration of disordered responses and corner-case behaviors in industrial cyber-physical systems. Third, we will investigate deeper integration with formal specification and verification techniques, so that property-driven test generation and distributed runtime monitoring can be combined with the proposed crowdsourced testing platform to provide stronger assurance for ICPS security and reliability. In parallel, we plan to refine the risk classification introduced in the Introduction based on feedback from real deployments, so that future test campaigns can be systematically aligned with the most critical ICPS risk categories.

## Figures and Tables

**Table 1:** Valid verification rate.

Method	Thread Count	Verification Rate for Different Delay	
		0 ms	1 ∼10 ms
Baseline	1	100 %	N/A
	2	73.91%	N/A
	4	54.05%	N/A
Apache JMeter	1	100%	N/A
	2	87.72%	N/A
	4	63.19%	N/A
Locust	1	100%	66.67%
	2	99.94%	66.67%
	4	98.86%	66.85%
Our Method	2	100%	100%
	4	100%	100%

## Data Availability

The original contributions presented in this study are included in the article. Further inquiries can be directed to the corresponding authors.
